# Microarray Study of Pathway Analysis Expression Profile Associated with MicroRNA-29a with Regard to Murine Cholestatic Liver Injuries

**DOI:** 10.3390/ijms17030324

**Published:** 2016-03-01

**Authors:** Sung-Chou Li, Feng-Sheng Wang, Ya-Ling Yang, Mao-Meng Tiao, Jiin-Haur Chuang, Ying-Hsien Huang

**Affiliations:** 1 Genomics and Proteomics Core Laboratory, Department of Medical Research, Kaohsiung Chang Gung Memorial Hospital and Chang Gung University College of Medicine, 833 Kaohsiung, Taiwan; raymond.pinus@gmail.com (S.-C.L.); wangfs@ms33.hinet.net (F.-S.W.); 2 Department of Anesthesiology, Kaohsiung Chang Gung Memorial Hospital and Chang Gung University College of Medicine, 833 Kaohsiung, Taiwan; yaling453@yahoo.com.tw; 3 Department of Pediatrics, Kaohsiung Chang Gung Memorial Hospital and Chang Gung University College of Medicine, 833 Kaohsiung, Taiwan; tmm@cgmh.org.tw; 4 Department of Surgery, Kaohsiung Chang Gung Memorial Hospital and Chang Gung University College of Medicine, 833 Kaohsiung, Taiwan; jhchuang@cgmh.org.tw

**Keywords:** miR-29a, bile duct ligation, cholestasis, liver fibrosis, TGF-β signaling pathway, Wnt signaling pathway

## Abstract

Accumulating evidence demonstrates that microRNA-29 (miR-29) expression is prominently decreased in patients with hepatic fibrosis, which consequently stimulates hepatic stellate cells’ (HSCs) activation. We used a cDNA microarray study to gain a more comprehensive understanding of genome-wide gene expressions by adjusting miR-29a expression in a bile duct-ligation (BDL) animal model. Methods: Using miR-29a transgenic mice and wild-type littermates and applying the BDL mouse model, we characterized the function of miR-29a with regard to cholestatic liver fibrosis. Pathway enrichment analysis and/or specific validation were performed for differentially expressed genes found within the comparisons. Results: Analysis of the microarray data identified a number of differentially expressed genes due to the miR-29a transgene, BDL, or both. Additional pathway enrichment analysis revealed that TGF-β signaling had a significantly differential activated pathway depending on the occurrence of miR-29a overexpression or the lack thereof. Furthermore, overexpression was found to elicit changes in Wnt/β-catenin after BDL. Conclusion: This study verified that an elevated miR-29a level could alleviate liver fibrosis caused by cholestasis. Furthermore, the protective effects of miR-29a correlate with the downregulation of TGF-β and associated with Wnt/β-catenin signal pathway following BDL.

## 1. Introduction

Hepatic inflammation and hepatitis caused by chronic cholestasis may lead to cirrhosis, which is a complicated process controlled by numerous signaling pathways and involves activation and hepatocyte apoptosis of a range of cells [1,2,3,4]. The hepatic stellate cells’ (HSCs) activation drives the fibrogenic process, which undergoes morphologic and functional trans-differentiation of the contractile myofibroblastic cells that contributes to excessive extracellular matrix (ECM) synthesis and their deposition in the affected hepatic tissues [1,2,5].

MicroRNAs (miRs) are small single-stranded RNAs consisting of 21–22 nucleotides, which interfere with gene expression through post-translational silencing RNA [6]. An increasing amount of research has shown that the expression of miR-29a, b, and c are decreased remarkably in liver fibrosis. These results are found not only in human liver cirrhosis, but also in mice with liver injury caused by bile duct ligation (BDL) and carbon tetrachloride (CCl4) intoxication [4,7]. We previously revealed that overexpression of miR-29a in cholestatic mice hindered the progression of liver fibrosis through a evident decrease in pro-apoptotic regulators and NF-κB , as well as a considerable increase in anti-apoptotic proteins, ultimately resulting in a considerable decrease in the extent of hepatocellular apoptosis [4]. Furthermore, miR-29a overexpression reduced HDAC4 actions, as well as HSC migration and propagation [3]. Since cholestatic liver injury consists of a number of complex processes, an innovative map of gene expression profiles regulated by miR-29a in animal models of cholestasis has yet to be researched and developed. Therefore, we used a cDNA microarray study to obtain a more comprehensive understanding of genome-wide gene expressions through adjustments of miR-29a expressions in BDL animal models and make such a map.

## 2. Results

### 2.1. Gain of miR-29a Signaling Considerably Mitigates Liver Fibrosis in Cholestatic Livers

**Figure 1:**
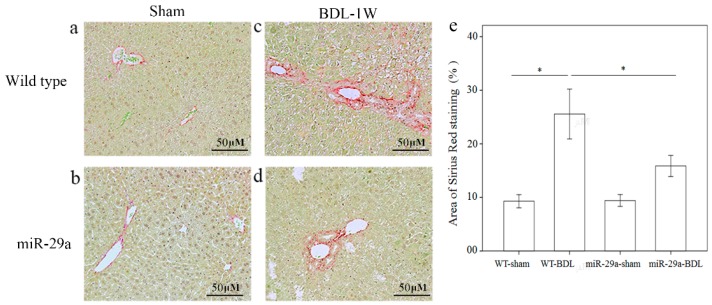
Overexpression of miR-29a in the murine model caused the downregulation of fibrosis in the mice livers following bile duct ligation. Sirius Red staining showed faint fibrosis in both sham operation of wide type (**a**) and miR-29a Tg mice (**b**) as well as moderate fibrosis in the –wild type mice (**c**) and mild fibrosis in the miR-29aTg mice (**d**), which was limited to the immediate vicinity of the portal area. (**e**) Data from the three samples per group are expressed as the mean ± SE. * indicates a *p* < 0.05 between the groups.

To determine the effects of miR-29a overexpression on the development of cholestatic liver injury, we investigated ECM production and deposition using Sirius Red staining. Figure 1 shows that moderate fibrosis was found in WT mice and mild fibrosis in miR-29aTg mice that was near the portal area after one week of BDL.

### 2.2. Analyses of Microarray Data

**Figure 2:**
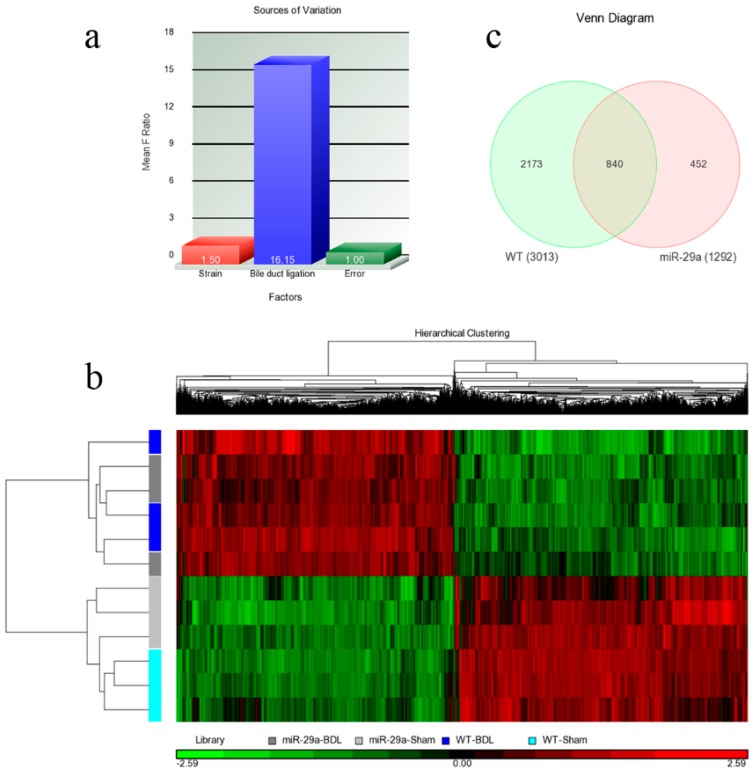
Overall gene expression changes. We used Agilent SurePrint G3 Mouse GE 8 × 60 K microarray chips to study the gene expressions. (**a**) The source of the variation plot shows that the bile duct ligation factor accounted for most of the variation; (**b**) The heat map illustrated the clustering of the sample sets; (**c**) WT denoted the WT-BDL *vs.* WT-sham comparison, while miR-29a denoted the miR-29a-BDL *vs.* miR-29a-sham comparison. The digits in the Venn diagram denote the number of DE genes in the set differences and their intersections.

Regarding each set of experimental animals, RNA samples were extracted from three mice, and the microarray samples were replicated three times. Therefore, two factors strain (miR-29a *vs.* WT) and bile duct ligation (BDL *vs.* sham) can all be found within the samples. Once the microarray experiments were performed, their raw data were analyzed using Partek with log2 transformation and quantile normalization. Among the strain and BDL factors, the latter resulted in much more variation than the former (Figure 2a), a phenomenon that was also observed in Figure 2b. The heat map reflects that the 12 samples were initially categorized based on their BDL factor. Within the sham or BDL sets, the samples cannot be further categorized into wild or miR-29a transgenic type with any clarity. Overall, BDL affected the experimental mice much more strongly than the miR-29a transgene. Since the miR-29a transgenic mice were found to have slightly higher miR-29a expressions [1], living organisms were determined to actually trend toward maintaining a homeostatic state and producing weaker alterations on gene expression profiles. Since miR-29a displayed protection ability against liver fibrosis, differentially expressed genes (DE) were then identified. After setting the criteria of FDR <0.05 and expression ratio >1.5, we collected DE genes for comparison. As shown in Figure 2c, we identified 3013 and 1292 genes that are differentially expressed within WT-BDL *vs.* WT-sham and miR-29a-BDL *vs.* miR-29a-sham comparisons, respectively. Furthermore, 840 genes were simultaneously differentially expressed within the two comparisons. By analyzing the DE genes in both models, we found considerably enriched pathways. Table 1 provides the significantly enriched pathways in the set difference of the WT-BDL *vs.* WT-Sham comparison, while Table 2 presents the significantly enriched pathways in the set difference of the miR-29a-BDL *vs.* miR-29a-Sham comparison.

### 2.3. Overexpression of miR-29a Considerably Hinders TGF-β Signaling Pathways in Cholestatic Livers

**Figure 3:**
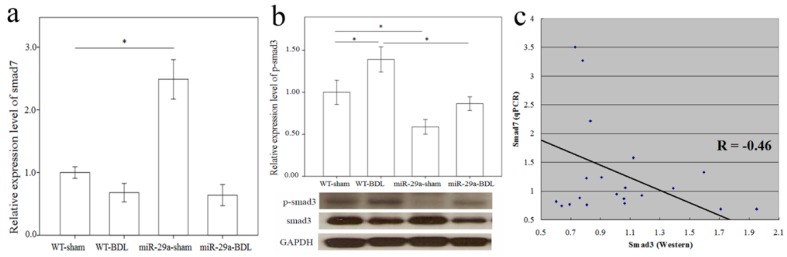
Comparison of the mRNA levels of Smad7 (**a**) and protein levels of Smad3 (**b**) expressions in the liver of WT and miR-29Tg mice following bile duct ligation (BDL). Western blot was adopted to study Smad3 activity; (**c**) We used a scatter plot to demonstrate that Smad7 and Smad3 negatively correlated with Pearson’s correlation coefficient (*R* = −0.46, *p* = 0.02). Data from the six samples per group are expressed as the mean ± SE. * indicates a *p* < 0.05 between the groups.

More and more research has shown that TGF-β is vital for liver fibrogenesis due to its function in HSC activation and ECM synthesis [8]. Consequently, TGF-β signaling pathway activation was observed in the WT mice after BDL (Table 1, *p* = 0.009). The overexpression of miR-29a notably caused the significant loss of activation of the TGF-β signaling pathway in livers after BDL. Compared to the DE genes of the TGF-β signaling pathways in WT mice after BDL, 10 up-regulated (shown in the red box) and five down-regulated (shown in the green box) genes were found (Figure S1). A negative regulator of the TGF-β signaling pathway, Smad7 protects the liver from fibrosis [9,10]. In the miR-29a transgenic mice, Smad7 maintained an abundance that was more than twice as great (miR-29a-sham *vs.* WT-sham, *p* < 0.001), thus providing evidence of its protection ability against liver fibrosis (Figure 3a). Nevertheless, because of the strong effects of cholestasis, Smad7 was significantly downregulated in the miR-29aTg mice with cholestasis (*p* < 0.001). Smad3 is known to have the role of a transcriptional activator of the TGF-β signaling pathway, thus promoting the liver fibrosis [11]. Since no significant difference was found in Smad3 mRNA expression in the microarray data, we studied protein levels using Western blotting. As Figure 3b shows, a significantly higher expression of phospho-Smad3 was observed in tissue from the BDL group than from the sham-operated group of the WT mice (*p* = 0.043). Instead, the miR-29aTg mice showed a considerably downregulated expression of phospho-Smad3 compared to that of their WT littermates (Figure 3b; *p* = 0.005). We subsequently investigated the association between Smad7 mRNA levels and phospho-Smad3 protein levels, and univariate analysis revealed that Smad7 mRNA levels negatively correlated with phospho-Smad3 protein levels (*R* = −0.46, *p* = 0.02) (Figure 3c).

### 2.4. Overexpression of miR-29a Considerably Increased Wnt/β-Catenin in Cholestatic Livers

**Figure 4:**
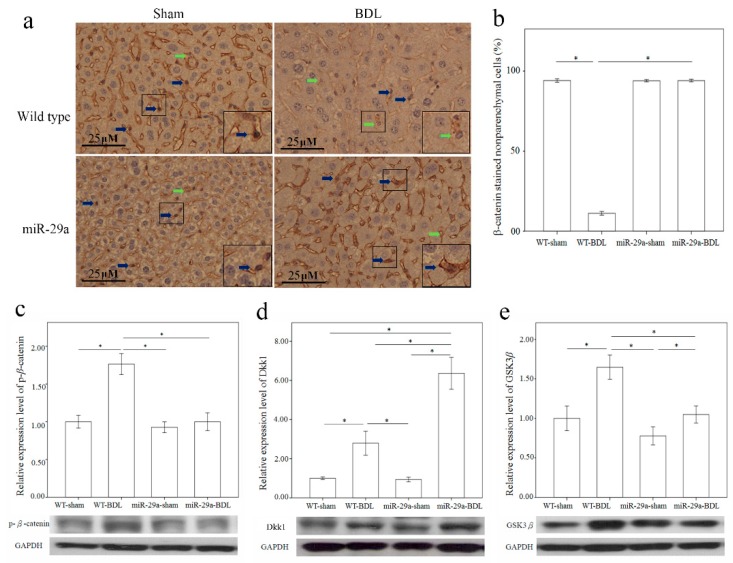
Overexpression of miR-29a in the murine model caused the upregulation of β-catenin (**a**,**b**), and Dkk1 (**d**) as well as the downregulation of phospho-β-catenin (**c**) and GSK3β (**e**) in the liver of mice following bile duct ligation. The β-catenin immunoreactivity staining mainly in cytoplasm of hepatocytes and nucleus of the non-parenchymal cells morphologically identical to HSC or Kupffer cells (Figure 4a, inset). Comparing to the sham group of WT mice, which was constantly expressed in the nucleus of the non-parenchymal cells (blue arrow), there was significantly lower β-catenin immunoreactivity (green arrow) in the liver tissues of the BDL group of WT mice (*p* < 0.001) (Figure 4a,b). Furthermore, miR-29a overexpression significantly upregulated the nuclear β-catenin immunoreactivity in miR-29aTg mice with cholestasis in comparison to WT littermates (*p* < 0.001). Data are expressed as mean ± SE (standard error) in the six samples from miR-29a transgenic mice and the six samples from their WT littermates. * indicates *p* < 0.05 between the groups.

Wnt/β-catenin pathway reportedly plays an important role in fibrotic diseases, including pulmonary, liver, skin, and renal fibrosis [12]. Thus, we used immunohistochemical staining to characterize β-catenin protein expression in the liver after BDL. Comparing to the sham group of WT mice, β-catenin was constantly expressed in the nucleus of the nonparenchymal cells morphologically identical to HSC or Kupffer cells, there was significantly lower β-catenin immunoreactivity in the liver tissues of the BDL group of WT mice (*p* < 0.001) (Figure 4a,b). Furthermore, miR-29a overexpression significantly upregulated the nuclear β-catenin immunoreactivity in miR-29aTg mice in comparison to WT littermates after BDL (*p* < 0.001). Phosphorylation of β-catenin reportedly escalates b-catenin ubiquitination and degration by proteasome pathways [13]. Therefore, we further measured β-catenin and phospho-β-catenin levels by Western blotting in the liver. As shown in Figure 4c, the BDL group of WT mice exhibited an increase in phospho-β-catenin protein expression (*p* < 0.001) in comparison with the sham-operation group, thus signifying that miR-29a may influence nuclear β-catenin expression during the early phases of cholestasis.

### 2.5. Overexpression of miR-29a Considerably Increased Dickkopf-Related Protein 1 (Dkk1) Expression in Cholestatic Livers

The secreted antagonists Dkk1 [14] and glycogen synthase kinase 3 β (GSK3β) [15,16] also regulate Wnt signals. Therefore, upon performing Western blotting analysis, we found a significantly increased expression of Dkk1 and GSK3β proteins in the liver from the BDL group than in those from the sham group (*p* = 0.004 and <0.001, respectively) in the WT mice (Figure 4d,e). Furthermore, miR-29a overexpression considerably upregulated Dkk1 protein expression (*p* < 0.001) in miR-29aTg mice in comparison to their WT littermates after BDL.

## 3. Discussion

This study delivers insight into a unique mechanism that determined that the overexpression of miR-29a in cholestatic mice considerably caused differentially expressed genes due to the miR-29a transgene, BDL, or both, as discovered by our microarray study. Pathway enrichment analysis found that TGF-β signaling had a significant differentially activated pathway with or without miR-29a overexpression. Furthermore, such overexpression of miR-29a resulted in changes in Wnt/β-catenin following BDL.

TGF-β is the most crucial mediating factor for fibrosis progression as it encourages HSC activation and ECM deposition [17]. TGF-β produced by hepatocytes, Kupffer cells, and sinusoidal endothelial cells is found to activate HSC trans-differentiation and increase ECM production [8]. Afterward, the activated HSC secretes profibrogenic mediators, such as TGF-β, and produces ECM components. Such a response is temporary if the injury is acute and reversible, but if the injury continues, the activated HSC will keep producing profibrogenic and ECM proteins that will ultimately compromise liver function [1,2,5]. Moreover, miR-29 overexpression in murine HSC caused the downregulation of collagen expressions, including col1α1 and col1α2 [7,18] through inhibiting mRNA expression of extracellular matrices. Since fibrosis represents a deregulation between ECM deposition and degradation, the miR-29–mediated suppression of ECM synthesis in HSCs could ideally counteract these excessive remodeling reactions that resulted in a low fibrosis activity *in vivo*. Smad7, a negative regulator of the COL1α2 gene, decreases at the time that collagen mRNA is being upregulated [19].

TGF-β1 signaling reportedly downregulates miR-29 expression in HSCs [7]. This reaction is also implicated in Bandyopadhyay *et al.*’s study [18]. Furthermore, Smad3 works as a transcriptional activator of the TGF-β signaling transduction that exacerbates hepatic fibrosis [11]. Through interacting with miR-29 promoter, Smad3 is observed to mediate TGF-β1-induced inhibition of miR-29 [20]. In contrast, our results revealed that miR-29aTg mice following BDL had a significant decrease in phospho-Smad3 level compared to their WT littermates. Consistent with our previous observation of the protective actions of miR-29 in liver fibrosis [3,4], miR-29 also acts as a potent fibrosis-inhibitory regulator that alleviates TGF-β1/Smad3-mediated renal fibrosis [21] and pulmonary fibrosis [22].

Wnt signaling transduction is found to control cell growth and differentiation [23]. Activating its canonical pathways suppresses GSK3β, protects β-catenin from degradation and subsequently facilitates the β-catenin translocation into the nucleus and thereby enhances the expression of cell cycle-regulators c-myc, c-jun, and cyclin D1 [15,16]. This study’s microarray data showed that cell cycle signaling pathways were significantly enriched in the WT gene set, but not in the miR-29a gene set. Moreover, Dkk1 is a potent Wnt antagonist [14]. Cheng *et al.* reported that canonical Wnt signaling is involved in the HSC activation. Wnt antagonism prevents HSC activation and liver fibrosis [24]. In an *in vitro* study, gain of Dkk1 expression is found to increase PPAR-γ expression but lower cell proliferation and type I collagen production in the HSC activation and eventually leads to HSC apoptosis [24]. In the meantime, fibrotic reactions in the liver can be reduced by Pokeweed antiviral protein, which is suggestive of a potent regulator detrimental to Wnt/β-catenin signaling. Increasing Pokeweed antiviral protein decreases the production and distribution of α-SMA [25]. In contrast, Kordes *et al.* showed that canonical Wnt signaling maintains the HSC quiescence [26]. Stimulation of Wnt/β-catenin signaling by TWS119 counteracts α-SMA expression but promotes GFAP synthesis [26]. Consistent with our analyses, miR-29a overexpression upregulated Dkk1 protein and maintained Wnt signaling in the quiescent stage of HSC hepatic stellate cells in the livers of the miR-29aTg mice with cholestasis in comparison to their WT littermates. However, the biological roles of β-catenin activation in the hepatic fibrosis warrants further investigation as the current evidence remains inconclusive.

In mouse glomeruli, extracellular TGF-β1 is found to change Wnt expression, activate β-catenin, and increase Wnt-responsive genes Snail1, MMP-7, MMP-9, desmin, Fsp1, and PAI-1 that participate in podocyte injury-mediated albuminuria [27]. Furthermore, TGF-β through Smad3 and p38MAPK pathways activates β-catenin in human dermal fibroblasts [28]. We have also shown that gain of miR-29a function ameliorated the glucocorticoid-induced disturbances of Wnt and Dkk1 actions and sustained osteoblast function and mineralization [29]. All together, these findings led us to hypothesize that miR-29a takes part in the interplay between Wnt/β-catenin and TGF-β signaling in the pathogenesis liver fibrosis. Since fibrosis represents a balance between ECM deposition and degradation, miR-29–mediated suppression of ECM synthesis in HSCs can ideally tip this balance that attenuates fibrosis reactions. Having insights into the genes actively responding to miR-29a signaling will be valuable to explore the therapeutic strategy of miR-29a signaling for improving liver fibrosis. The emerging marker for fibrosis progression sheds a light on the fibrogenic progression of hepatic tissues.

The limitation of current study is that we do not address all the other DE signal pathways that are changed in the miR-29a protection of liver fibrosis. Their biological function is worthy of characterization in the future.

## 4. Materials and Methods

### 4.1. Ethics Statement

The Institutional Animal Care and Use Committee (IACUC) of Kaohsiung Chang Gung Memorial Hospital approved the animal use protocol (#2012090301). Twelve-week-old male FVB mice (body weight 23–25 g) were procured from BioLASCO Taiwan Co., Ltd. (Taipei, Taiwan). All animals were housed in a facility (air condition at 22 °C and relative humidity of 55%), a 12 h light/12 h dark cycle, sterile rodent chop, and tap water *ad libitum*.

### 4.2. Greation of miR-29a Transgenic Mice

Gene sequences that coded PGK promoter and miR-29a precursor were, respectively, cloned using PCR protocols. The clones of interest were subjected to insertion into pUSE plasmid, and the linear PGK-miR-29a-BGH poly-A were further cloned and purified for microinjection. Fertilized eggs from FVB/N mice were transferred with the designed construct and then transplanted into Crl: CD1 foster mothers, as previously described [3,30]. Transgenic mice that carried the construct of interest were verified by PCR and primers (forward: 5’-GAGGATCCCCTCAAGGAT ACCAAGGGATGAAT-3’ and reverse 5’-CTTCTAGAAGGAGTGTTTCTAGGTATCCGTCA-3’) [19,29] and accommodated in a specific pathogen-free rodent barrier.

### 4.3. Animal Model and Experimental Protocol

Animals received a ligation of common bile duct were categorized as “BDL” group, and those received sham ligation were served as “sham” group. Anesthesia and surgical procedures for ligation were performed in aseptic conditions, as previously described [4]. At 1 week postoperation, animals were subjected to euthanasia. Liver tissues were dissected, snap frozen, and stored at −80 °C for extracting total RNA, tissue lysates, and biochemical analysis.

### 4.4. Histological Analysis

Specimens were subjected to embedding in a TissueTek^®^ optimal cutting temperature (OCTTM) compound (Sakura Finetek) in liquid nitrogen and stored at −80 °C. Four μm thick frozen sections were prepared with a Leica CM3050-S cryostat system (Leica, Wetzlar, Germany) for Sirius Red staining. Briefly, sections were fixed in 4% paraformaldehyde (Sigma-Aldrich, Saint Louis, MO, USA), incubated in 1% BSA (Sigma-Aldrich, Saint Louis, MO, USA), and then stained by Sirius Red (Sigma-Aldrich, Saint Louis, MO, USA), according to the manufacturer’s instructions. For immunohistochemical staining, sections from paraffin-embedded archival liver specimens were probed by β-catenin antibody (sc-7963, Santa Cruz Biotechnology, Dallas, TX, USA), and secondary antibody of a SuperPicTure^TM^Polymer detection kit (Zymed Laboratories, Francisco, CA, USA), according to the manufacturer’s manual. Sections probed by buffer only were used as negative controls. Immunostaining intensity of sections was measured by independent color channel of an image J analysis.

### 4.5. Microarray Samples

Total RNA in snap-frozen hepatic tissues was isolated with REZOLTMC&T agent (Protech Technology, Taipei, Taiwan), according to the manufacturer’s instructions. A total of 0.2 μg RNA was amplified by Low Input Quick-Amp Labeling Kit (Agilent Technologies, Santa Clara, CA, USA) and labeled with Cy3 (Agilent Technologies, Santa Clara, CA, USA). Aliquots of 0.6 μg of the Cy3-labled cRNA were mixed with a fragmentation buffer and incubated at 60 °C for 30 min. The fragmented Cy3-labled cRNAs were subjected to hybridization into Agilent SurePrint G3 Mouse GE 8 × 60 K Microarray Chips (Agilent Technologies, Santa Clara, CA, USA), according to the manufacturer’ instructions. Fluorescence Cy3 signals in the chips were detected with an Aligent microarray scanner setting at 535 nm. Cys signals in each scanned images were quantified by a Feature Extraction10.5.1.1 image analysis software (Agilent Technologies, Santa Clara, CA, USA) normalized with background signal. The RNA samples of 12 mice were obtained from the following four sets of mice: miR-29a-BDL, miR-29a-sham, WT-BDL, and WT-sham.

### 4.6. mRNA Expression Detected by Real-Time Quantitative RT-PCR

Quantification of mRNA expression was performed by an ABI 7700 Sequence Detection System (TaqMan; Applied Biosystems, Waltham, MA, USA). For reverse transcription, aliquots of total RNA were reacted with 2× TaqMan^®^ PreAmp Master Mix and 10× Megaplex™ PreAmp Primers. The templates were further reacted with 2× TaqMan^®^ Universal PCR Master Mix and primers for PCR amplification. Comparative threshold cycle (*C*_t_) was measured by the ABI7700 system. Relative mRNA expression were calculated by 2^−(Δ*C*t target − Δ*C*t calibrator)^ or 2^−ΔΔ*C*t^. PCR amplicons were further verified by 2% agarose electrophoresis (AMRESCO, Solon, OH, USA). Specific primers Smad7 (forward, 5’-AAACTACTTGCTGCTAACCT-3’; Reverse, 5’-GGACACACGGATGACGA-3’) and GAPDH (forward, 5’-CACTGCCACCCAGAAGA-3’; reverse, 5’-TCCACGACGGACACATT-3’) were designed. We repeated the validation experiments in order to confirm the amplification efficiencies. Six animals were used.

### 4.7. Immunoblotting

Aliquots of 30 µg protein extracts were subjected to SDS-PAGE electrophoresis. Proteins on the gels were blotted onto a nitrocellulose membrane. Proteins of interest on the blots were, respectively, probed by GSK3β (#9315, Cell signaling, Danvers, MA, USA), Dkk1 (sc-25516, Santa Cruz Biotechnology, Dallas, MA, USA), GAPDH (GTX100118, GeneTex, Irvine, CA, USA), phospho-Smad3 (p-Smad3; ab51451, abcam, Cambridge, UK), Smad 3 (ab40854, abcam, Cambridge, UK), phospho-β-catenin (p-β-catenin ; sc-16743-R, Santa Cruz Biotechnology, Dallas, MA, USA), and β-catenin (sc-7963, Santa Cruz Biotechnology, Dallas, MA, USA). After washing, the blots were further incubated with secondary antibodies horseradish peroxidase-coupled anti-mouse immunoglobulin-G antibodies. The proteins designated were visualized by an enhanced chemiluminescence detection (GE Healthcare Biosciences AB, Little Chalfont, UK). Intensities of protein bands were quantified by a gel densitometry system. Six animals were used for detection.

### 4.8. Statistical Analysis

All of the values are expressed as mean ± standard error. Data was analyzed with one-way analysis of variance [30] and the least significant difference (LSD) test was used for *post-hoc* testing. Relationships between quantitative variables were analyzed by Pearson’s coefficient. A *p* value less than 0.05 were considered statistically significant. Differentially expressed (DE) genes underwent pathway enrichment analysis with Partek from Partek Incorporated (Saint Louis, MO, USA). *p*-values were determined using the hypergeometric test.

## 5. Conclusions

Through this study, we verified that elevation of miR-29a alleviates liver fibrosis caused by cholestasis. Furthermore, the protective potential of miR-29a is linked to the downregulation of TGF-β and is associated with Wnt signaling pathways.

## Figures and Tables

**Table 1:** Results of pathway enrichment analysis on the 2173 genes differentially expressed in the WT-BDL *vs.* WT-Sham comparison alone.

Pathway Name	*p* Value	# of Genes in the Pathway	Pathway ID
Metabolic pathways	0.000001	145	kegg_pathway_235
Peroxisome	0.000229	19	kegg_pathway_157
Cell cycle	0.000548	24	kegg_pathway_53
Retinol metabolism	0.000743	16	kegg_pathway_57
Steroid hormone biosynthesis	0.000904	14	kegg_pathway_294
Protein digestion and absorption	0.001179	16	kegg_pathway_206
Arachidonic acid metabolism	0.002021	15	kegg_pathway_38
Biosynthesis of antibiotics	0.002177	31	kegg_pathway_274
Lysine degradation	0.003177	13	kegg_pathway_203
Fatty acid degradation	0.004790	11	kegg_pathway_125
TGF-β signaling pathway	0.009331	15	kegg_pathway_93
Linoleic acid metabolism	0.010929	8	kegg_pathway_263
Amoebiasis	0.011037	18	kegg_pathway_111
Tryptophan metabolism	0.012916	9	kegg_pathway_88
Progesterone-mediated oocyte maturation	0.014029	16	kegg_pathway_200
ECM-receptor interaction	0.015281	14	kegg_pathway_147
Chemical carcinogenesis	0.018625	12	kegg_pathway_229
Butanoate metabolism	0.025619	6	kegg_pathway_248
Bile secretion	0.027883	12	kegg_pathway_291
Oocyte meiosis	0.029539	17	kegg_pathway_230
Small cell lung cancer	0.030371	14	kegg_pathway_190
PPAR signaling pathway	0.033269	13	kegg_pathway_78
Regulation of lipolysis in adipocytes	0.037877	10	kegg_pathway_255
Histidine metabolism	0.038958	5	kegg_pathway_165
Drug metabolism—other enzymes	0.039071	8	kegg_pathway_36
Fatty acid metabolism	0.046776	9	kegg_pathway_27
Fatty acid biosynthesis	0.048322	4	kegg_pathway_89
Mineral absorption	0.049102	8	kegg_pathway_236

**Table 2:** Results of pathway enrichment analysis on the 452 genes differentially expressed in the miR-29a-BDL *vs.* miR-29a-Sham comparison alone.

Pathway Name	*p* Value	# of Genes in the Pathway	Pathway ID
Steroid hormone biosynthesis	0.000021	8	kegg_pathway_294
Leukocyte transendothelial migration	0.000029	11	kegg_pathway_276
Natural killer cell mediated cytotoxicity	0.000347	10	kegg_pathway_253
Chemical carcinogenesis	0.000356	7	kegg_pathway_229
Fc epsilon RI signaling pathway	0.000914	7	kegg_pathway_100
Platelet activation	0.001147	9	kegg_pathway_23
B cell receptor signaling pathway	0.001389	7	kegg_pathway_65
Osteoclast differentiation	0.002132	9	kegg_pathway_135
Arrhythmogenic right ventricular cardiomyopathy (ARVC)	0.002642	6	kegg_pathway_1
Retinol metabolism	0.002727	6	kegg_pathway_57
Bile secretion	0.002771	6	kegg_pathway_291
Phagosome	0.003856	10	kegg_pathway_194
Hypertrophic cardiomyopathy (HCM)	0.005919	6	kegg_pathway_279
Energy metabolism	0.011458	8	kegg_pathway_16
Pentose and glucuronate interconversions	0.021195	3	kegg_pathway_285
Metabolic pathways	0.021733	31	kegg_pathway_235
Focal adhesion	0.022124	9	kegg_pathway_187
Inositol phosphate metabolism	0.022784	4	kegg_pathway_49
Viral carcinogenesis	0.024087	9	kegg_pathway_216
Axon guidance	0.025580	7	kegg_pathway_264
Antigen processing and presentation	0.025778	5	kegg_pathway_269
Viral myocarditis	0.026037	5	kegg_pathway_47
Linoleic acid metabolism	0.027978	3	kegg_pathway_263
Dilated cardiomyopathy	0.029280	5	kegg_pathway_277
Autoimmune thyroid disease	0.039891	4	kegg_pathway_64
Biosynthesis of antibiotics	0.040668	8	kegg_pathway_274
Fc gamma R-mediated phagocytosis	0.041882	5	kegg_pathway_120
2-Oxocarboxylic acid metabolism	0.046164	2	kegg_pathway_35
HTLV-I infection	0.047823	10	kegg_pathway_189

## Supplementary Materials

Supplementary materials can be found at http://www.mdpi.com/1422-0067/17/3/324/s1.
