# Supplementary material for: Microarray Study of Pathway Analysis Expression Profile Associated with MicroRNA-29a with Regard to Murine Cholestatic Liver Injuries
Source: Int J Mol Sci. 2016 Mar 1;17(3):324. doi: 10.3390/ijms17030324 (PMC4813186; doi:10.3390/ijms17030324)
Supplement: Supplementary file 1 [file ijms-17-00324-s001.pdf]

Supplementary Materials: Microarray Study of Pathway Analysis Expression Profile Associated with MicroRNA-29a with Regard to Murine Cholestatic Liver Injuries

Sung-Chou Li, Feng-Sheng Wang, Ya-Ling Yang, Mao-Meng Tiao, Jiin-Haur Chuang and Ying-Hsien Huang

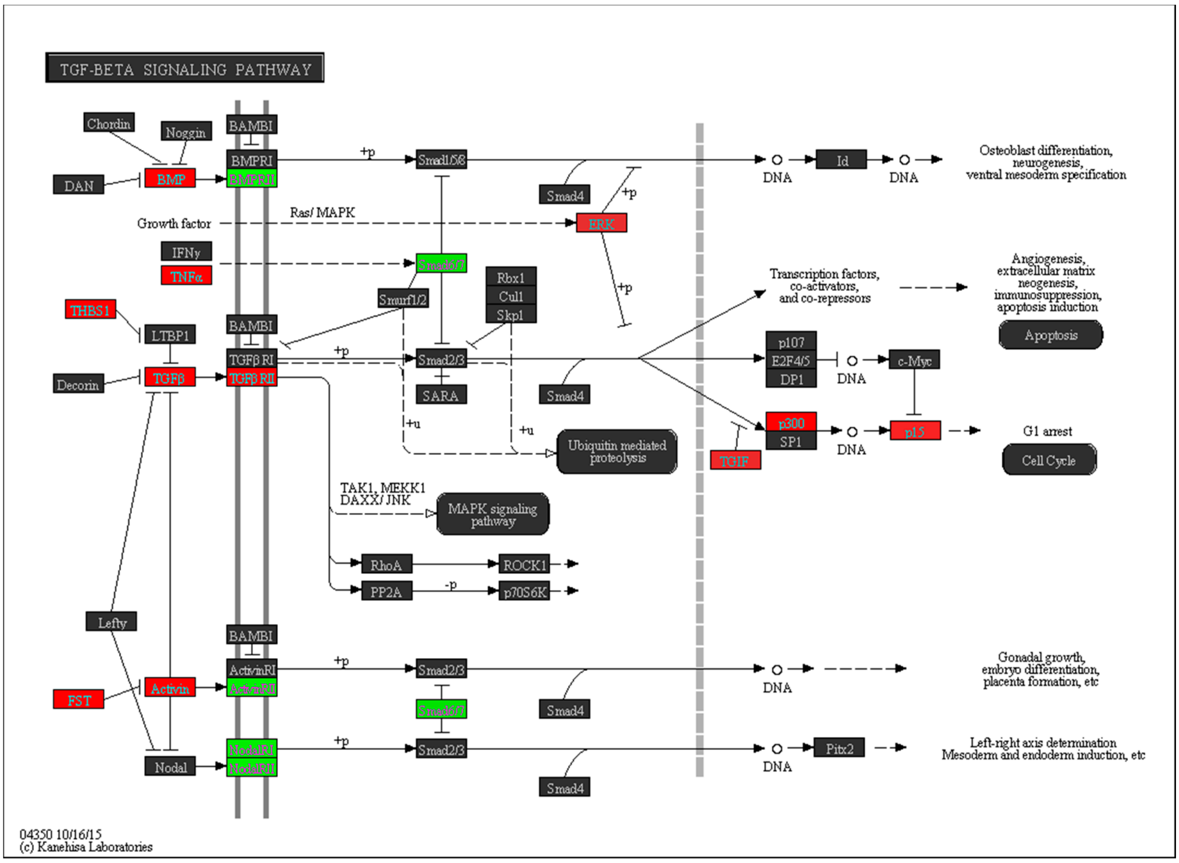

Figure S1. TGF-β signaling pathway. We mapped the differentially expressed genes back to KEGG pathways to determine if they were significantly enriched in several pathways. The TGF-beta signaling pathway was significantly enriched (p = 0.009331) by 2173 differentially expressed genes within the WT-BDL *vs.* WT-sham comparison. The genes that underwent upregulation, downregulation, and non-alteration in the BDL samples are highlighted in the red, green, and gray boxes, respectively.
